# Case report: Efficacy of later-line fam-trastuzumab deruxtecan in a patient with triple-positive breast cancer with brain metastases

**DOI:** 10.3389/fonc.2024.1470560

**Published:** 2024-11-04

**Authors:** Ingrid Karmane Sumou, Cheng Vai Hui

**Affiliations:** Department of Oncology, Centro Hospitalar Conde de São Januário, Macao, Macao SAR, China

**Keywords:** antibody-drug conjugate, triple-positive breast cancer, human epidermal growth factor receptor 2 (HER2), brain metastasis, fam-trastuzumab deruxtecan

## Abstract

Fam-trastuzumab deruxtecan (T-DXd) has demonstrated substantial antitumor activity and durable responses in patients with human epidermal growth factor receptor 2 positive (HER2+) metastatic breast cancer. We report here the treatment outcomes of T-DXd in a patient with HER2+ breast cancer with brain metastases that repeatedly recurred and progressed after two lines of salvage therapy. In 2016, a 23-year-old G0P0 female with risk factors including menarche at age 9 years, Li-Fraumeni syndrome, and a strong family history of cancer was diagnosed with bilateral, triple-positive breast cancer. She received chemotherapy, HER2-targeted therapies, total mastectomy, and locoregional radiotherapy, but a brain metastasis in the left parieto-occipital lobe was detected in 2020. After receiving capecitabine, lapatinib, gonadotropin-releasing hormone (GnRH) agonist, and tamoxifen, multiple new lesions appeared in the brain after 14 months. The patient then received capecitabine, neratinib, GnRH agonist, and letrozole; however, her brain metastases still progressed after 7 months. In 2022, she started T-DXd treatment. Good response to treatment was observed 4 months later, including a continuous decrease in the cancer antigen 15-3 level, a reduction in the size of the major brain tumor, and the absence of new lesions. Now aged 30, the patient is continuing to receive T-DXd treatment to prevent recurrence. We conclude that T-DXd was effective for the treatment of brain metastases in this young patient with triple-positive metastatic breast cancer who had multiple risk factors and had received several anti-HER2 therapies prior to T-DXd.

## 1 Introduction

Breast cancer (BC) is the most common cancer worldwide (1), and BC tumors that overexpress human epidermal growth factor receptor 2 (HER2) comprised about 14% of all new cases in the USA from 2016–2020 (2). Although HER2+ tumors tend to be more aggressive than other BCs, the prognosis varies depending on the co-expression of hormone receptors (HRs), namely estrogen receptor (ER) and progesterone receptor (PR), and patients with triple-positive BC tumors tend to be younger (≤ 49 years) (3). Among patients with advanced BC, approximately 25% developed brain metastasis, with the number climbing to 30–40% in those with HER2-positive tumors (4). Treatments targeted at HER2, which include monoclonal antibodies, antibody-drug conjugates (ADCs), and small-molecule tyrosine kinase inhibitors (TKIs), have led to greatly improved survival in patients with HER2+ tumors (4).

The current standard of care for patients in the metastatic setting is first-line dual-HER2 monoclonal antibody therapy with pertuzumab and trastuzumab, plus a taxane (5, 6). Globally, patients with unresectable or metastatic HER2+ BC that progressed on ≥ 2 prior therapies also now have the option of the antibody-drug conjugate fam-trastuzumab deruxtecan (T-DXd) (7). T-DXd consists of a HER2-targeted antibody and a cleavable, membrane-permeable topoisomerase I inhibitor that is preferentially released inside of cancer cells (8). In the DESTINY-Breast01 trial of heavily pretreated patients with unresectable or metastatic HER2+ BC, T-DXd provided an objective response rate (ORR) of 62%, an 18.2-month (95% confidence interval [CI], 15.0 months to not evaluable) duration of response, and a small (1.6%) rate of further disease progression (7). A subgroup analysis (9) showed that the efficacy of T-DXd was also durable in those with brain metastases (N = 24), with a confirmed ORR of 58.3% (95% CI, 36.6–77.9) and a median progression-free survival (PFS) of 18.1 months (95% CI, 6.7–18.1). In a subsequent trial (DESTINY-Breast02) of a similar patient population, T-DXd was compared with treatment of physician’s choice (capecitabine plus either trastuzumab or lapatinib) (10). This trial confirmed the positive benefit–risk profile of T-DXd, which showed a median PFS of 17.8 months (vs. 6.9 months in the other group; hazard ratio [HR], 0.36; 95% CI, 0.28–0.45; P < 0.0001) over a median follow-up of 21.5 months. Furthermore, in the prospective phase 2 TUXEDO-1 trial of 15 patients with HER2+ BC and active brain metastases, treatment with T-DXd led to a high intracranial response rate (73.3%; 95% CI, 48.1–89.1) (11). In the retrospective ROSET-BM study (12), T-DXd was also shown to have promising efficacy in HER2+ BC patients with active brain metastases as well as leptomeningeal carcinomatosis, which are associated with poor prognosis and remain difficult to treat (4, 13).

In this report, we detail the case of a 30-year-old woman with triple-positive BC who developed brain metastases that was successfully treated with third-line T-DXd. The reporting of this case conforms to the CARE guidelines (14). Written informed consent was obtained from the patient to publish this paper.

## 2 Case description

### 2.1 Diagnosis of triple-positive breast cancer with multiple risk factors

Our patient, a G0P0 female, was diagnosed with bilateral, triple-positive invasive ductal carcinoma (Ki-67 index range: 45–80%) at the age of 23 in January 2016. Pre-treatment positron emission tomography (PET) and magnetic resonance imaging (MRI) detected a multicentric lesion (4.0 cm × 4.5 cm) at the 2H position of the left breast, with left axillary lymph node metastases (level I–III), and two lesions (2.8 cm × 2.1 cm at 8H and 2.4 cm × 1.8 cm at 10H) in the right breast (**Table 1**). The tumor in the left breast was clinically staged as IIIA cT2N2M0, and those in the right breast were staged as IIA cT2(m)N0M0. Genetic analysis identified a *TP53* mutation (possibly germline), suggesting the possibility of Li-Fraumeni syndrome. Individuals with this syndrome have a significantly increased risk of developing breast cancer and are recommended to undergo frequent oncological monitoring (15). Our patient tested negative for pathogenic variants of *BRCA1*, *BRCA2* and *PTEN*, but reported a strong family history of cancers that included nasopharyngeal, liver, and colon cancers among the maternal and paternal grandparents. She also experienced early menarche (age, 9 years), which is a well-established risk factor for breast cancer (16).

**Table 1:** Timeline and course of treatment for the patient.

Diagnosis	Treatment	Date
Primary bilateral invasive ductal carcinoma with lymph node involvement	Neoadjuvant paclitaxel 80 mg/m^2^ IV weekly for 16 weeks; trastuzumab 8 mg/kg (loading) IV for first cycle, followed by 6 mg/kg Q3W; pertuzumab 840 mg (loading) IV for first cycle, followed by 420 mg Q3W; leuprolide 11.25 mg IM Q3M	Feb 2016
	Bilateral nipple-sparing total mastectomy, left axillary dissection, right sentinel node biopsy, implant insertion	Aug 2016
	Locoregional RT; trastuzumab 6 mg/kg Q3W + pertuzumab 420 mg Q3W for up to 1 year; leuprolide 11.25 mg IM Q3M; letrozole 2.5 mg orally daily	Nov 2016
Second primary RT-induced sarcoma of chest wall	Wide local excision with clear margins	Oct 2018
Metastatic brain lesions from breast primary	Tumor resection (7 x 5 cm dura-based lesion in left parieto-occipital lobe); local and whole-brain RT; capecitabine 1000 mg b.i.d. for day 1–14 Q3W (2 weeks on, 1 week off); lapatinib 750 mg daily for first week, followed by 1250 mg daily thereafter; leuprolide 11.25 mg IM Q3M; tamoxifen 10 mg b.i.d.	Jun 2020
Metastatic bone and recurrent brain lesions	Corpectomy; capecitabine 1000 mg b.i.d. for day 1–14 Q3W (2 weeks on, 1 week off); neratinib 120 mg daily for 2 weeks, then 160 mg daily for 1 week, followed by 240 mg daily thereafter; leuprolide 11.25 mg IM Q3M; letrozole 2.5 mg orally daily; denosumab 120 mg SC Q4W	Nov 2021; Mar 2022; Apr 2022
Progression of brain metastases	T-DXd 5.4 mg/kg Q3W; antiemetics and corticosteroids as needed	Nov 2022–Ongoing

### 2.2 Treatment of breast primary

In February 2016, the patient was treated with weekly paclitaxel, HER2-targeted monoclonal antibody therapies (trastuzumab + pertuzumab), and gonadotropin-releasing hormone (GnRH) agonist for 16 weeks. All of the masses became non-palpable after treatment. A post-neoadjuvant chemotherapy PET scan showed metabolic quiescence in both breasts and axilla. In August 2016, she underwent a bilateral nipple-sparing total mastectomy, left axillary dissection, right sentinel node biopsy, and implant insertion. Pathology revealed the following: no residual tumors in the left tumor bed at 2H (ypT0N1M0) and the right tumor bed at 8H; residual invasive ductal carcinoma grade II (0.2 cm with 3 foci), with no lymphovascular invasion, a posterior margin of 1 cm, and associated ductal carcinoma *in situ* in the right tumor bed at 10H, scored as ypT1N0(sn)(i-)M0, with triple positivity (ER Allred score of 8, PR Allred score of 6, and HER2 immunohistochemistry [IHC] score 3+) and a Ki-67 index of 7%; and involvement of 3/20 left axillary nodes (largest: 0.8 cm with 0.3 cm extracapsular extension), but no involvement among 4 sentinel nodes in the right axilla. The patient received adjuvant locoregional radiotherapy (RT; 40 Gy in 15 fractions) of the left supraclavicular fossa and the left chest in November 2016. She was maintained for up to 1 year on anti-HER2 therapy with trastuzumab and pertuzumab, with a GnRH agonist and letrozole.

### 2.3 Brain metastasis from breast primary and second primary malignancy

**Figure 1:**
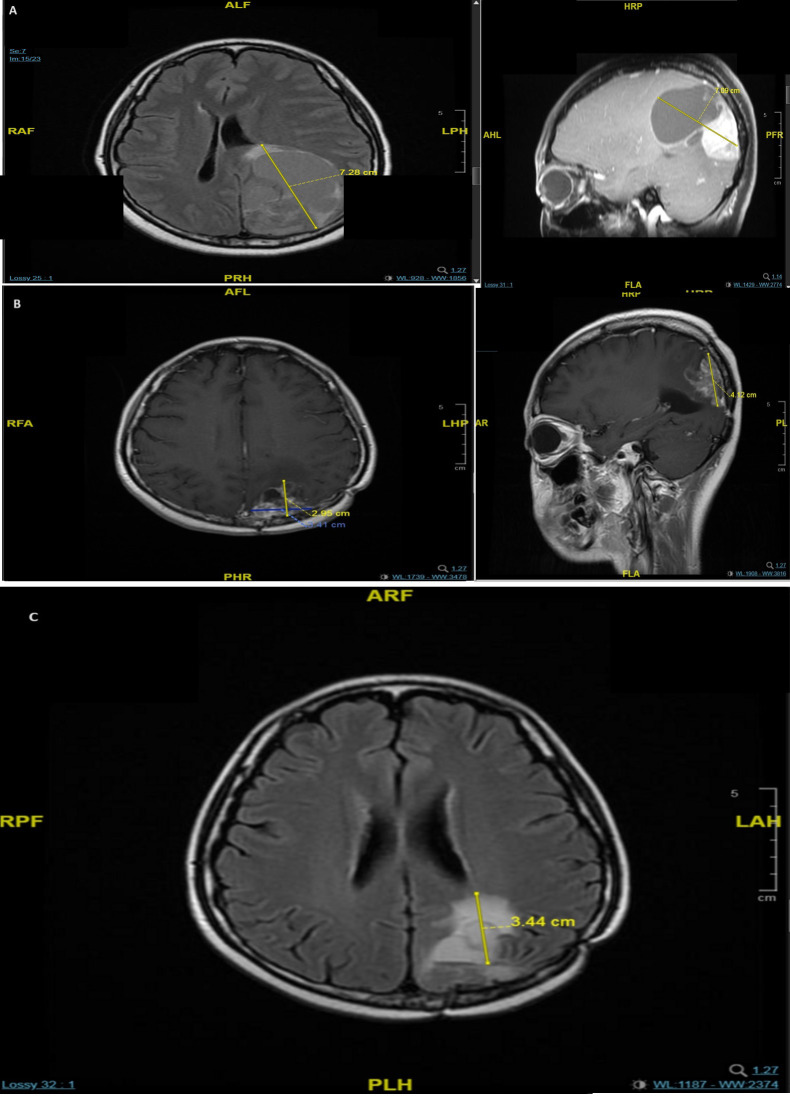
MRI scans of brain metastases in the patient showing a 7 × 5 cm dural-based tumor in the left parieto-occipital lobe in May 2020 **(A)**, recurrence with multiple lesions in the left parieto-occipital region in December 2021 **(B)**, and progression of the major lesion in the left parieto-occipital region in November 2022 **(C)**.

In October 2018, she was diagnosed with a second primary malignancy: a RT-induced undifferentiated pleomorphic sarcoma (1.1 cm) of the left chest wall, which was treated with wide local excision with clear margins. Eighteen months later, she presented with headache and diplopia, and received an MRI scan that showed a firm, dural-based tumor (7.0 cm × 5.0 cm) with hypervascularity in the left parieto-occipital lobe (**Figure 1A**). Tumor resection revealed pathology consistent with metastatic BC. She completed RT (40 Gy in 10 fractions) for the brain tumor bed and whole-brain RT (30 Gy in 10 fractions). In August 2020, she was given capecitabine, lapatinib, a GnRH agonist, and tamoxifen as first-line treatment for the metastatic BC.

Follow-up computed tomography (CT) and MRI scans conducted between October 2020 and March 2021 showed no evidence of tumor recurrence. Considering the risk of colorectal cancer associated with Li-Fraumeni syndrome, two colonoscopies were conducted (April 2019 and April 2021), neither of which showed any significant findings.

### 2.4 Management of recurrences of brain metastases

In November 2021, an MRI showed multiple bone metastases at the L1, L3, L5, and S1 vertebrae. The patient was given denosumab 120 mg subcutaneously every 4 weeks (upon the drug approval from the hospital committee since April 2022). The L3 segment had spinal canal invasion, with pathological fracture and spinal stenosis, which was treated with a corpectomy. The pathological results indicated metastatic BC remained to be triple positive (ER 5, PR 0, HER2 IHC score 3+, and a Ki67 index of 25%). A brain MRI scan in the following month showed recurrence, with multiple lesions in the left parieto-occipital region (largest: 3.4 cm × 2.9 cm × 4.1 cm) (**Figure 1B**). In March 2022, the patient received second-line treatment comprising capecitabine plus targeted therapy with neratinib, a GnRH agonist, and letrozole (her menses stopped in October 2021). A follow-up brain MRI scan 1 month later showed a partial response, and a CT scan in October 2022 showed stable disease. However, after 7 months of neratinib treatment, MRI showed disease progression of the left parieto-occipital brain lesion (**Figure 1C**). We initiated third-line treatment with T-DXd (5.4 mg/kg once every 3 weeks) in November 2022. T-DXd has proven its superiority over chemotherapy in late lines, and for closer observation of adverse effects, hormonal therapy was saved sequentially after the chemo-containing ADC.

**Figure 2:**
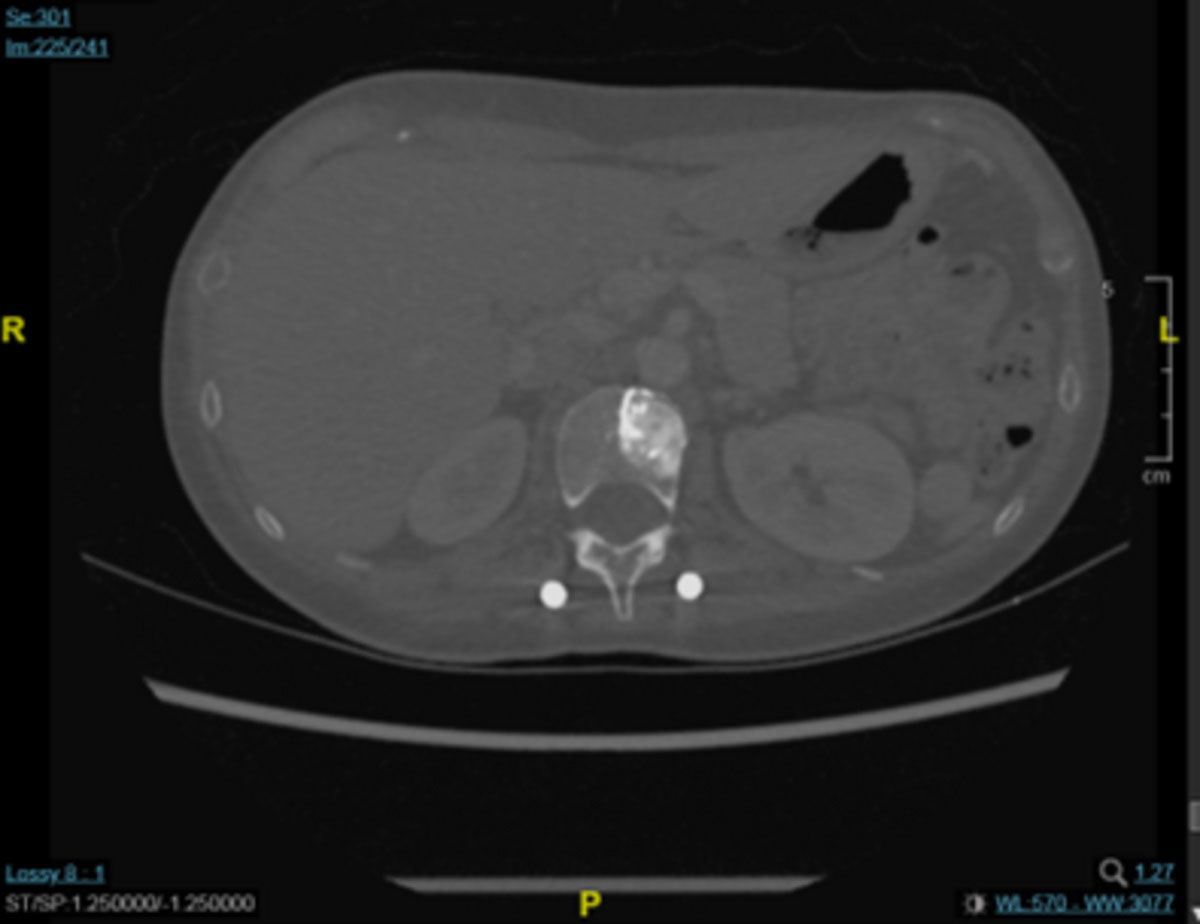
Chest CT scan of the T2 vertebral metastasis in March 2023.

**Figure 3:**
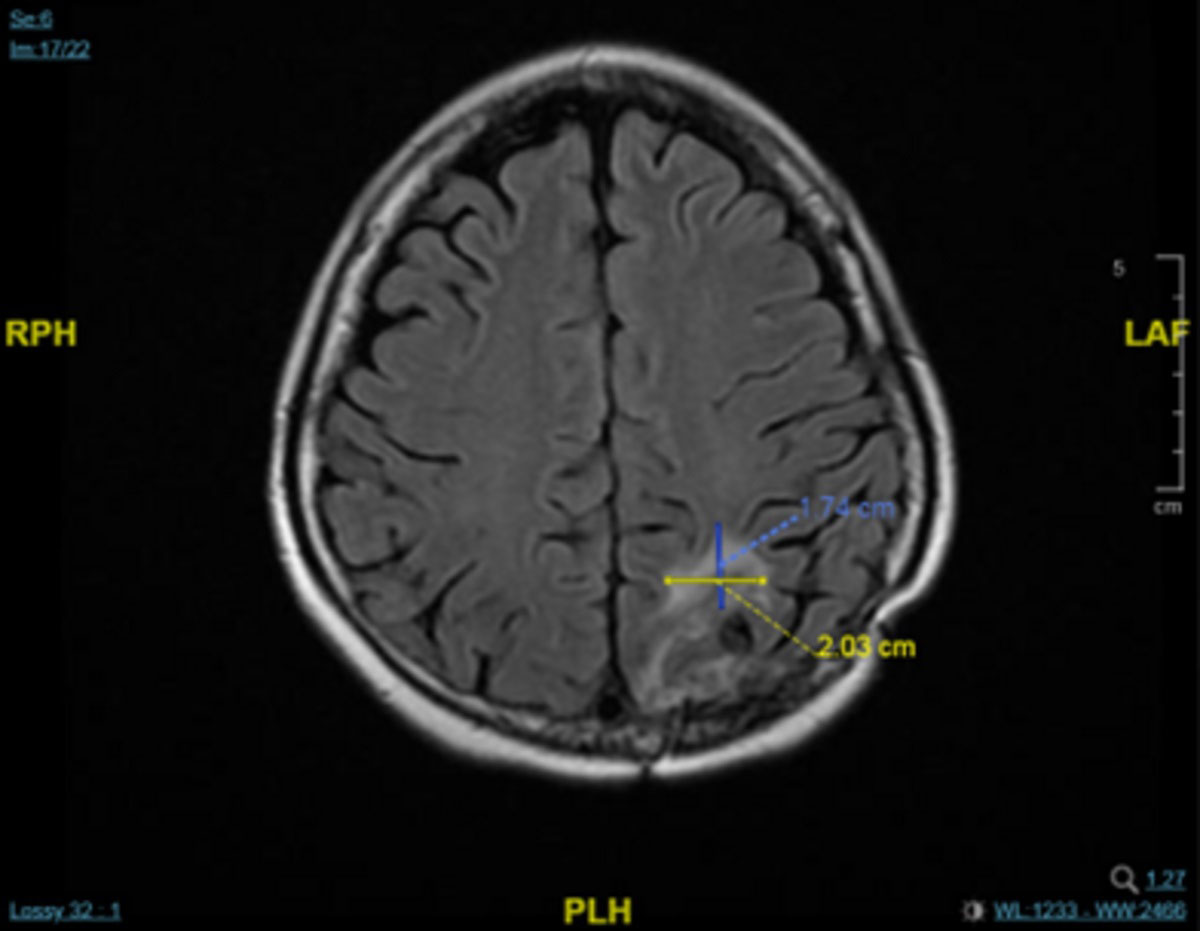
MRI scan showing a decrease in the mass in left parieto-occipital lobe after treatment with T-DXd in April 2023.

A chest CT scan 4 months later showed that there had been no progression in the T2 vertebra, in which remained a round osteoblastic lesion (1.5 cm × 1.5 cm; **Figure 2**). This CT scan also showed some fibrous stripes in the upper lobe of the left lung, but no other abnormalities were observed. Furthermore, monitoring of the cancer antigen 15-3 (CA15-3) level showed a continuous decrease from 48.5 U/mL to 11.2 U/mL between December 2022 and April 2023. Brain MRI showed that the size of the major mass in the left parieto-occipital lobe had decreased from 3.7 cm × 3.0 in November 2022 to 2.0 cm × 1.7 cm in April 2023 (**Figure 3**). Several cysts and small lesions remained in the left parieto-occipital region; however, no new lesions or midline shifts were found, and the ventricles and basal cisterns appeared normal. We concluded that the brain metastases responded well to treatment with T-DXd, and the patient has since been asymptomatic. Follow up chest and abdomen CT scans in July 2023 and October 2023 consistently showed post-surgical change of the lumbar spine in stable status with no new visceral or bony lesions.

The patient remains on T-DXd at the time of this writing in November 2023. To manage the potential side effects of T-DXd, supportive measures including antiemetics and corticosteroids were provided. The patient also received nursing and health education related to the new systemic therapy, as well as exercise and diet counseling. During follow-up visits while on therapy, the patient reported that she was able to maintain her professional and personal activities, including continuing her teaching career and volunteering at a cancer patient group to help other patients. From the perspective of quality of life, the treatment not only helps the patient achieve intact everyday functioning, but also enables her to actively participate in the community.

## 3 Patient perspective

T-DXd was accessible and effective for the treatment of brain metastases with triple-positive metastatic BC who had multiple risk factors and received multiple anti-HER2 therapies.

## 4 Discussion

For what used to be a tumor subtype with an aggressive phenotype bearing high recurrence rates and very poor outcomes, HER2+ advanced BC now beholds more treatment options. The introduction of HER2-directed therapies, most notably, trastuzumab, pertuzumab, ADCs such as trastuzumab emtansine (T-DM1) and T-DXd, and TKIs such as lapatinib, neratinib, pyrotinib, and tucatinib, has shown improvements in the prognosis of patients with HER2+ metastatic BC.

T-DXd has shown magnificent activity in patients pretreated with T-DM1. In the DESTINY-Breast01 trial of 184 heavily pretreated patients with a median of 6 prior lines of therapy, T-DXd provided a median PFS of 16.4 months. Responses were observed in 112 patients (60.9%; 95% CI, 53.4–68.0); the disease control rate was 97.3% (95% CI, 93.8–99.1) (17). These groundbreaking data resulted in the U.S. Food and Drug Administration (FDA) granting accelerated approval to T-DXd for patients with HER2+ cancer who have received 2 or more prior anti-HER2-targeted therapies in the metastatic setting (18).

Tucatinib is another agent with proven efficacy when given as third-line treatment for HER2+ advanced BC. Based on results from the phase 2 HER2CLIMB trial (19), the FDA approved tucatinib for use with trastuzumab and capecitabine in patients with unresectable or metastatic HER2+ BC, including those with brain metastases, who have been pretreated with at least 1 prior HER2-directed regimen in the metastatic setting (20). According to the trial, patients benefited with a statistically significant improvement in ORR (40.6% [95% CI, 35.3–46.0] vs. 22.8% [95% CI, 16.7–29.8]; P < 0.001), median PFS (7.8 vs. 5.6 months; HR, 0.54; 95% CI, 0.42–0.71; P < 0.001) and median overall survival (OS; 21.9 vs. 17.4 months; HR, 0.66; 95% CI, 0.50–0.88; P = 0.005) (19). Patients with brain metastases also experienced an improvement in intracranial PFS (9.9 vs. 4.2 months; HR, 0.32; 95% CI, 0.22–0.48; P < 0.0001), intracranial ORR (47.3% [95% CI, 33.7–61.2] vs. 20.0% [95% CI, 5.7–43.7]; P = 0.03), and OS (18.1 vs. 12.0 months; HR, 0.58; 95% CI, 0.40–0.85; P = 0.005) (21). Nevertheless, in real world setting it all comes down to accessibility and socio-economics, with disparity in drug approvals and reimbursement strategies across different countries.

Neratinib in combination with capecitabine was approved for HER2+ advanced or metastatic BC following 2 or more anti-HER2-based regimens. In the phase 3 NALA trial, 621 eligible patients were randomized to receive either neratinib plus capecitabine or lapatinib plus capecitabine. The 12-month PFS rate was higher with use of the neratinib combination (28.8%; 95% CI, 23.1–34.8) than with the lapatinib combination (14.8%; 95% CI, 10.3–20.1), but there was no significant improvement in the 12-month OS rate (HR, 0.881; 95% CI, 0.72–1.07; P = 0.2086), with almost twice as common Grade 3 diarrhea (24.4% vs. 12.5%) (22). The limited clinical benefits plus the high toxicity make this agent less appealing when compared with other existing options approved by the FDA.

Margetuximab, an anti-HER2 monoclonal antibody, was approved for use with chemotherapy in patients with metastatic HER2+ BC following 2 or more HER2-directed therapies, with at least 1 being for metastatic disease. In the phase 3 SOPHIA trial, 536 eligible patients were randomized to receive margetuximab or trastuzumab. Margetuximab, designed to elicit an antibody-drug-mediated cellular cytotoxicity response, reached a statistically significant yet clinically modest improvement in median PFS (5.7 vs. 4.4 months; HR, 0.71; 95% CI, 0.58–0.86; P < 0.001), with improved ORR (22% vs. 16%; P = 0.06) (23). However, it appears only to work in patients who have a particular genotype that puts them at a higher risk of not being able to generate that response. This agent was locally inaccessible, plus we do not have a way to test for that genotype.

In view of the relatively large molecular size, ADCs were considered unlikely to penetrate the blood-brain barrier (BBB) and thus inefficacious for the treatment of brain metastases (24). However, prior RT to the brain tumor bed and whole-brain RT might induce leakage of the BBB at the site of metastases through apoptosis and senescence in cells of the neurovascular unit (25). Additionally, RT might lead to vascular leakage and further permeabilize the BBB by reducing astrocytes, pericytes, and tight junction proteins in paracellular channels (25), allowing T-DXd to penetrate the brain parenchyma and act on the tumors.

In the phase 2 TUXEDO-1 trial, T-DXd provided a high intracranial response rate (73.3%; 95% CI, 48.1–89.1) among patients with active brain metastases from HER2+ BC (11). Our patient also demonstrated the efficacy and safety of T-DXd for the treatment of brain metastases. After development and progression of brain lesions, our patient was started on T-DXd as third-line treatment and had a rapid reduction in the size of the major brain mass, with a significant decline in CA15-3 within the first 4 months. She found T-DXd well-tolerated and reported no serious adverse events.

T-DXd can be given as a second-line treatment in patients with metastatic HER2+ BC. In the phase 3 DESTINY-Breast03 trial (26), T-DXd significantly improved PFS (28.8 vs. 6.8 months; HR, 0.33; 95% CI, 0.26–0.43; P < 0.0001) and OS both not reached [NR] (HR, 0.64; 95% CI, 0.47–0.87; P = 0.0037) compared with T-DM1 in the second-line setting. The benefit of T-DXd over T-DM1 (PFS, 15.0 vs. 3.0 months; HR, 0.25; 95% CI, 0.13–0.45; ORR, 67.4% vs. 20.5%) was consistently demonstrated in the subgroup of patients with brain metastases (27). The European Society for Medical Oncology has recommended T-DXd as a second-line treatment option for patients with HER2+ BC (28).

## Data Availability

The original contributions presented in the study are included in the article/supplementary material. Further inquiries can be directed to the corresponding author.
